# Short‐Term Versus Long‐Term Efficacy of Endoscopic Sleeve Gastroplasty in 8880 Obese Patients: A Systematic Review and Meta‐Analysis

**DOI:** 10.1111/dom.70988

**Published:** 2026-06-18

**Authors:** Mohammed Barghash, Matthew Riad, Yasser Khalil, Khaled Mohamed, Ahmed Elgamasy, Omar Aly, Mohanad Abdelfattah, Mohamed Ibrahim Elgohary, Zeyad M. Wesh, Thilo Sprenger, Hany Takla, Amr Elserafy, Ahmed Abdelsamad

**Affiliations:** ^1^ North Bristol Foundation NHS Trust Bristol UK; ^2^ Faculty of Medicine Assiut University Assiut Egypt; ^3^ Medical Intern at Faculty of Medicine Helwan University Cairo Egypt; ^4^ General Surgery Resident at Cairo University Hospitals Cairo University Cairo Egypt; ^5^ Faculty of Medicine Cairo University Cairo Egypt; ^6^ Faculty of Medicine Port Said University Port Said Egypt; ^7^ Cairo University Hospitals Cairo Egypt; ^8^ Faculty of Medicine Newgiza University Cairo Egypt; ^9^ Faculty of Medicine Alexandria University Alexandria Egypt; ^10^ Head of the Bariatric Surgery Department Recklinghausen Germany; ^11^ Orlando Health Weight Loss and Bariatric Surgery Institute Orlando Florida USA; ^12^ Department of Trauma and Emergency General Surgery University Hospitals Birmingham NHS Foundation Trust Birmingham UK; ^13^ Department of Surgery II University of Witten/Herdecke Herdecke Germany; ^14^ Deputy Head of the Oncological Surgery Department, Section Head of Robotic Surgery Knappschaft Vest‐Hospital Recklinghausen Germany

**Keywords:** bariatric endoscopy, comorbidity remission, endoscopic sleeve gastroplasty (ESG), long‐term outcomes, obesity, short‐term outcomes, systematic review and meta‐analysis, weight loss

## Abstract

**Background:**

Endoscopic sleeve gastroplasty (ESG), a natural orifice transluminal endoscopic procedure, is increasingly integrated into multidisciplinary obesity care. We performed a systematic review and meta‐analysis to quantify the durability of ESG‐associated weight loss and metabolic outcomes across short‐ and long‐term follow‐up.

**Methods:**

Databases were searched from inception to September 2, 2025 (PROSPERO: CRD420251139333). Peer‐reviewed studies reporting ESG outcomes were included; primary outcomes were % total body weight loss (%TBWL), % excess body weight loss (%EBWL), and BMI reduction, stratified by follow‐up (≤ 6 months vs. ≥ 1 year). Random‐effects models were used; heterogeneity (*I* ^2^), publication bias (Egger's test), GRADE assessment, sensitivity analyses, and meta‐regression for baseline BMI were performed.

**Results:**

Thirty‐three studies (8880 patients; 31 cohorts, 2 RCTs) were included. %TBWL increased from 8.66% (1 month) to 16.13% (6 months) and was 17.88% at 1 year. Long‐term pooled %TBWL declined modestly over time (17.88% at 1 year to 15.67% at 3 years). Remission rates were 62% for type 2 diabetes, 43% for hypertension, and 43% for dyslipidaemia. Weight regain occurred in 6%. Baseline BMI predicted higher %TBWL but lower %EBWL.

**Conclusion:**

ESG is associated with clinically meaningful weight loss maintained beyond 6 months with modest attenuation over longer follow‐up, alongside favourable metabolic remission rates and low reported weight regain.

**Abbreviations**
%EBWL: percentage of excess body weight loss
%TBWL: percentage of total body weight loss
BMI: body mass index
CI: confidence interval
DOI: digital object identifier
GRADE: grading of recommendations assessment, development, and evaluation
IQR: interquartile range
IRB: institutional review board
LOHS: length of postoperative hospital stay
LOO: leave‐one‐out sensitivity analysis
MD: means difference
MeSH: medical subject headings
No.: number
NOS: Newcastle–Ottawa Scale
OR: odds ratio
PRISMA: preferred reporting items for systematic reviews and meta‐analyses
PROSPERO: The International Prospective Register of Systematic Reviews
RCT: randomized controlled trial
RoB2: Cochrane Risk of Bias 2.0 tool for Randomized Trials
SD: standard deviation
SRMA: systematic review and meta‐analysis
Supp.: supplementary
Vs.: versus

## 1 Introduction

Endoscopic sleeve gastroplasty (ESG) has emerged as an essential modality within contemporary obesity care, addressing a rapidly expanding disease burden that is tightly linked to cardiometabolic morbidity, impaired quality of life, and escalating healthcare costs [1, 2]. Despite the effectiveness of metabolic surgery, access remains constrained by limited capacity, patient compliance toward operative interventions, and the need for scalable therapies that can be deployed earlier in the disease course [3, 4]. ESG occupies an acceptable position in this landscape: it is a natural orifice, organ‐sparing, and repeatable, offering a pragmatic bridge between lifestyle/pharmacologic therapy and bariatric surgery, particularly for patients seeking a less invasive, yet durable intervention [5].

Beyond its procedural challenge, ESG is clinically relevant because it targets the core pathophysiology of obesity through restrictive gastric remodelling and downstream neurohormonal and behavioural effects, promoting portion control and sustained caloric reduction without the anatomical bypass of the small intestine [6, 7]. In real‐world practice, ESG is increasingly integrated into multidisciplinary pathways that include structured nutritional follow‐up, behavioural therapy, and adjunct pharmacotherapy [8]. As these integrated strategies gain headway, understanding the magnitude and durability of ESG‐induced weight loss, as well as its impact on metabolic comorbidities, becomes essential for evidence‐based treatment selection and patient counselling [9].

Long‐term efficacy is the critical benchmark that determines whether ESG can be considered more than a short‐lived “starter” procedure. While short‐term outcomes are commonly reported and often favourable, obesity is a chronic, relapsing condition in which weight trajectories, adherence to follow‐up, and physiological adaptation evolve over years rather than months. Physicians, therefore, require high‐quality longitudinal evidence to answer pragmatic questions: Does ESG maintain meaningful weight loss beyond the first postoperative year? How do outcomes change over time? What is the expected durability of metabolic benefits, and how frequently do patients experience weight regain or require additional interventions? [10].

Existing literature has been limited by heterogeneous reporting of follow‐up timepoints, variable definitions of outcomes and complications, and a predominance of single‐center observational cohorts [11, 12, 13]. These factors complicate interpretation and hinder the translation of findings into guideline‐level recommendations, reimbursement decisions, and standardized clinical pathways. Moreover, as ESG adoption has accelerated and techniques as well as peri‐procedural care have matured, contemporary evidence is needed to reflect current practice patterns and to provide effective estimates that are representative of today's patient selection, suturing strategies, and structured aftercare [3].

We conducted a systematic review and meta‐analysis to evaluate the long‐term durability of ESG, focusing on weight loss trajectories across short‐ and long‐term follow‐up. We pooled estimates for percent total body weight loss, percent excess body weight loss, and BMI reduction, and assessed additional domains including early adverse events, metabolic comorbidity remission, and weight regain. Given expected variability across endoscopic studies, we also explored heterogeneity and examined the influence of baseline BMI using meta‐regression. By synthesizing the most up‐to‐date evidence, we aimed to clarify the sustained clinical effectiveness of ESG and support its positioning within treatment algorithms, shared decision‐making, and standardized follow‐up pathways.

## 2 Methods

### 2.1 Protocol and Reporting Guidelines

The protocol for this review was registered in The International Prospective Register of Systematic Reviews (PROSPERO) [14] with reference number *CRD420251139333*. This systematic review followed the guidelines dictated by the Cochrane Collaboration and the Preferred Reporting Items for Systematic Reviews and Meta‐Analyses [15] (PRISMA‐2020) [16] (Table S1).

### 2.2 Search Strategy

**FIGURE 1:**
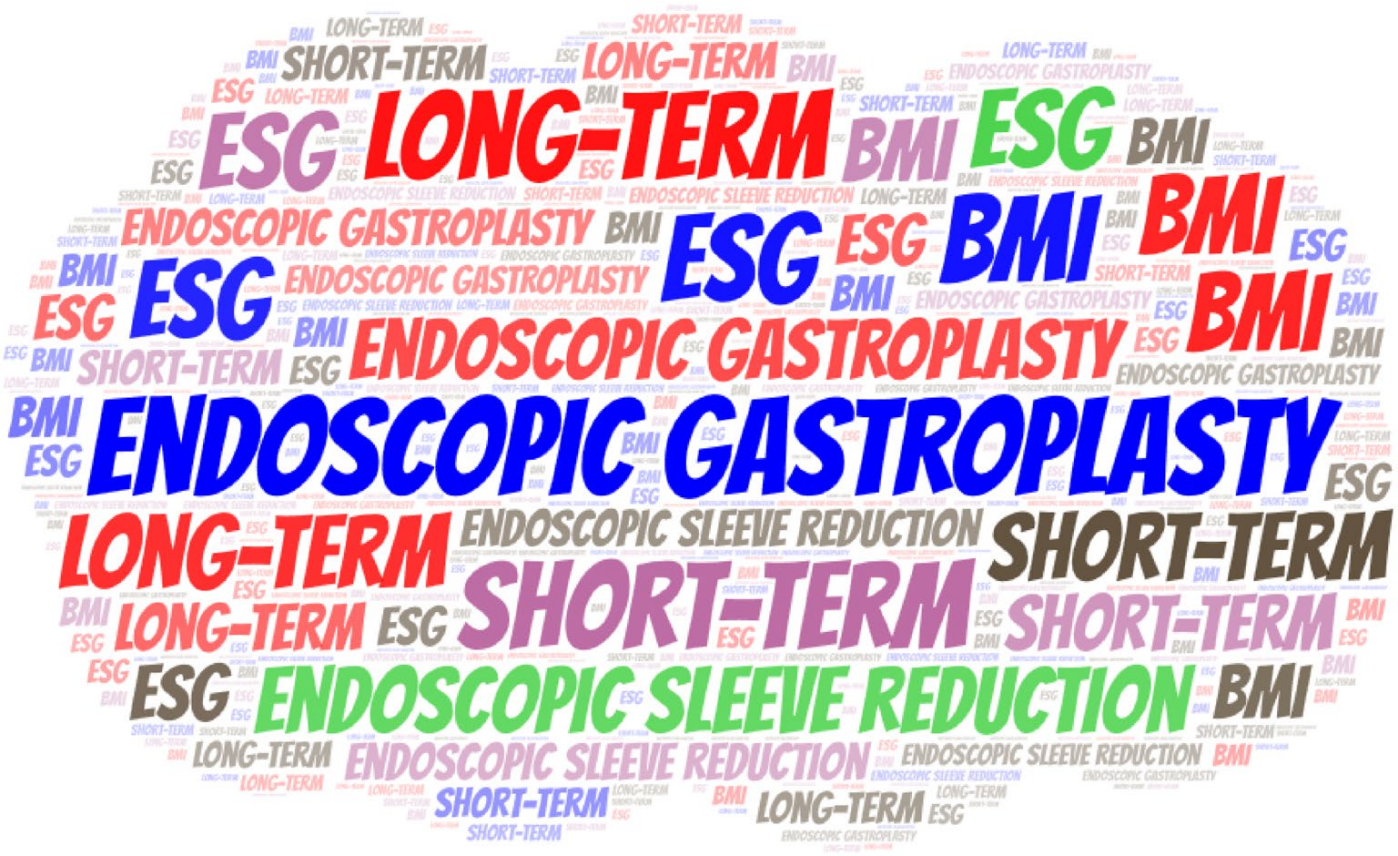
Word cloud.

A comprehensive literature search was conducted in PubMed, Scopus, and Web of Science to identify all relevant studies published up to September 2, 2025. The search strategy was developed using Medical Subject Headings (MeSH) and relevant terms related to ESG. To maximize sensitivity and ensure inclusivity, the search included a wide spectrum of keywords and synonyms related to ESG (including “endoscopic sleeve gastroplasty” OR “endoscopic gastroplasty” OR “endoscopic stomach sleeve” OR “endoscopic bariatric suturing” OR “endoscopic suturing system” OR “endoscopic plication system” OR “endoluminal gastric plication” OR “endoluminal gastroplasty”). See Figure 1 for a word cloud of the search strategy.

### 2.3 Selection Criteria

Our search focused on full‐text studies involving human subjects with obesity undergoing endoscopic sleeve gastroplasty (ESG). Eligible studies evaluated short‐term outcomes up to 6 months post‐procedure as well as long‐term outcomes of 1 year follow‐up or longer. These time intervals were selected to reflect the most consistently reported follow‐up periods across the literature. We excluded case reports, animal studies, systematic reviews and meta‐analyses, expert opinions, commentaries, abstracts, and editorials.

### 2.4 Screening and Data Extraction

Eligible studies were exported to Excel sheets for a two‐phase screening process: initial title and abstract review, followed by full‐text review. Three independent reviewers (M.R., A.E., and O.A.) conducted all assessments, with a fourth reviewer (A.A) resolving disagreements.

### 2.5 Study Variables and Outcomes

The primary outcomes included percentage of total body weight loss (%TBWL), percentage of excess body weight loss (%EBWL), and body mass index (BMI) reduction. Additional primary outcomes included procedure‐related complications, such as postoperative bleeding, postoperative epigastric pain, vomiting, and long‐term weight regain (defined as any increase in body weight after reaching nadir weight or regain of > 20% of the initially lost weight during follow‐up periods of approximately 4–12 months). Remission of obesity‐related comorbidities, including type 2 diabetes mellitus, hypertension, and dyslipidaemia, was also assessed.

%TBWL, %EBWL, and BMI reduction outcomes were stratified by follow‐up duration to assess differences between short‐term (≤ 6 months) and long‐term (≥ 1 year) outcomes after ESG. The secondary outcomes included: length of postoperative hospital stay (LOHS), re‐admission, re‐operation, and mortality rates.

### 2.6 Quality Assessment

Two independent reviewers (O.A. and M.A.) evaluated the quality of each included study in a blinded manner, using standardized tools, with any disagreements being addressed by consulting with a third reviewer (A.A). For cohort studies, the Newcastle‐Ottawa Scale (NOS) was applied. The scale covers three domains—Selection, Comparability, and Outcome—and awards a maximum of nine stars. Studies are classified into high quality (7–9 stars), moderate quality (4–6 stars), and low quality (0–3 stars) [17]. Randomized Controlled Trials (RCTs) were assessed using the Cochrane Risk of Bias 2.0 tool for randomized trials (RoB2), which evaluates five domains: randomization process, deviations from intended interventions, missing outcome data, measurement of outcomes, and selective reporting. Each domain is judged as low risk, some concerns, or high risk of bias [18].

### 2.7 Certainty of Evidence

The certainty of evidence of our statistical analysis results was assessed using the Grading of Recommendations Assessment, Development and Evaluation (GRADE) framework [19]. Randomized controlled studies were initially considered “high certainty” and then evaluated accordingly. Two independent reviewers were responsible for this, with disputes being settled by a third reviewer.

### 2.8 Statistical Analysis

A subgroup meta‐analysis of means was conducted for primary outcomes reported as continuous variables. Continuous outcomes were extracted as means and standard deviations (SDs); when results were reported as medians and interquartile ranges (or ranges), they were converted to means and SDs using the method proposed by McGrath et al. [20]. An analysis of proportions was performed for primary outcomes reported as dichotomous variables. All analyses were conducted using R‐Studio (version 2025.09.2, Build 418) with the *meta* package [21].

For continuous primary outcomes, including %TBWL, %EBWL, and BMI reduction, subgroup analyses were performed according to follow‐up duration. Differences between subgroups were assessed using the chi‐squared (*χ* ^2^) test for subgroup differences [22]. For dichotomous primary outcomes, such as postoperative complications and remission of comorbidities, pooled proportions were calculated. Funnel plots and Egger's test [23] were used to assess the potential publication bias.

Results were presented using forest plots, which illustrated individual study estimates, subgroup pooled estimates, and overall pooled estimates, along with corresponding 95% confidence intervals (CIs). A random‐effects model [24] was applied for all analyses. Statistical heterogeneity was assessed using the *I* ^2^ statistic, with values of 25%, 50%, and 75% indicating low, moderate, and high heterogeneity, respectively [25]. Leave‐one‐out sensitivity analyses were performed to explore potential sources of heterogeneity. A *p*‐value < 0.05 was considered statistically significant.

Some studies reported outcomes at multiple time points. Therefore, the same patient cohorts contributed data to different follow‐up subgroups. These data were treated separately to describe weight loss trajectories over time. As such, the results belong partially to overlapping populations. For this reason, short‐ versus long‐term comparison should be interpreted as descriptive rather than fully independent.

For each outcome, leave‐one‐out sensitivity analyses were conducted by sequentially omitting one study at a time from the corresponding meta‐analysis and re‐estimating the pooled effect size to assess the influence of individual studies on the overall results and observed heterogeneity; significant findings were reported under the respective outcomes. Forest plots of the sensitivity analyses can be seen in Figures S6–S8.

## 3 Results

**FIGURE 2:**
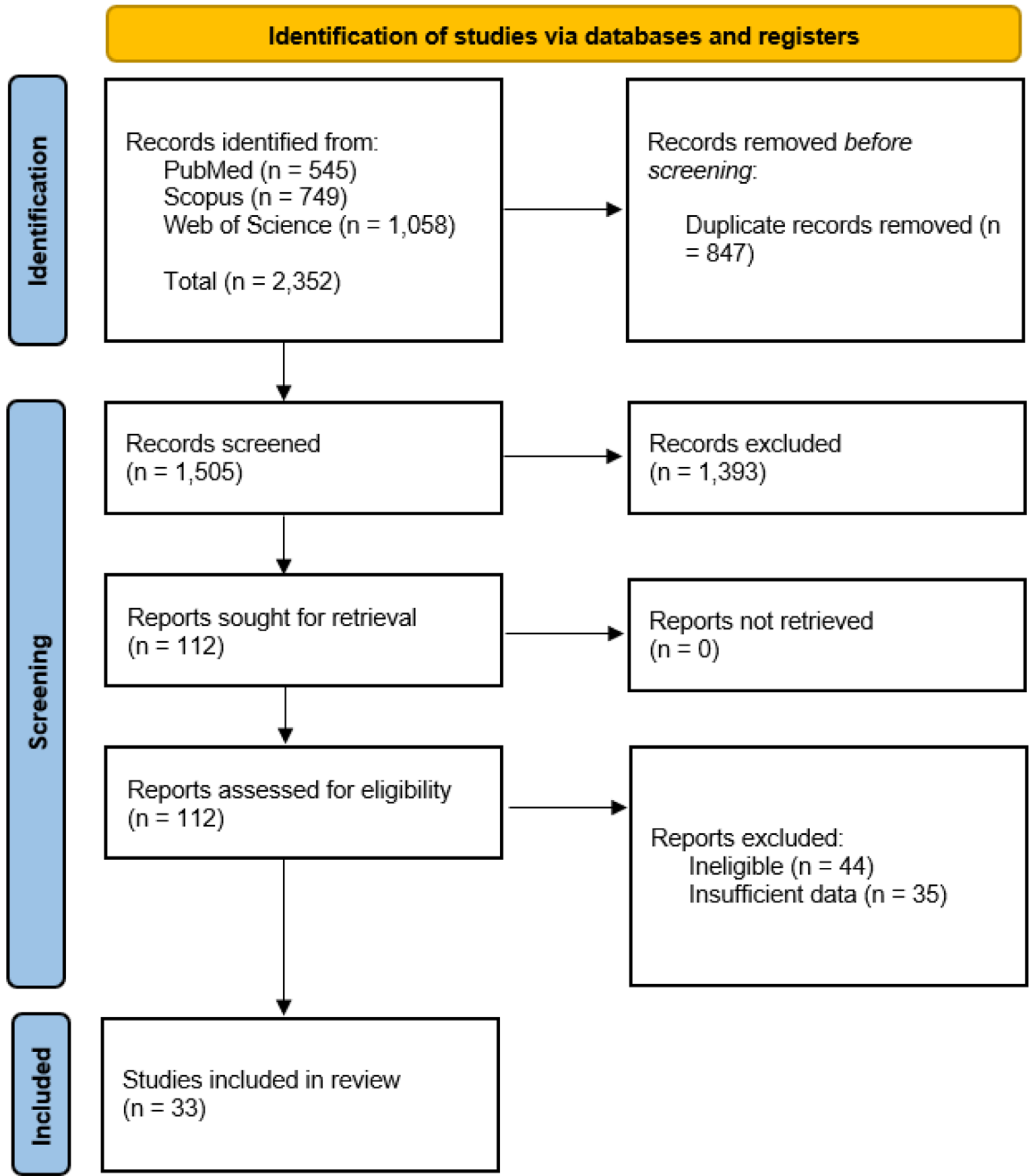
PRISMA flow diagram.

Following the database search, a total of **2352** records were identified. After screening and eligibility assessment, **33** studies, comprising **8880** class I‐III obese patients, met the inclusion criteria and were included in the meta‐analyses, as illustrated in the PRISMA flow diagram (Figure 2) [2, 10, 13, 26, 27, 28, 29, 30, 31, 32, 33, 34, 35, 36, 37, 38, 39, 40, 41, 42, 43, 44, 45, 46, 47, 48, 49, 50, 51, 52, 53, 54, 55]. Of the included studies, **31** were observational cohort studies, and **2** were randomized controlled trials (RCTs) [51, 55]. The studies and patients' baseline characteristics are presented in Tables 1 and 2, respectively. The DOI for each study is presented in Table S2.

**TABLE 1:** Study characteristics.

Study ID	Country	Study design	Study period
Abad 2025	Spain	Prospective multicenter, randomized, controlled, double‐blinded clinical trial (RCT)	April 2018 to December 2020
Alqahtani 2022	Saudi Arabia	Retrospective score–matched comparative cohort	December 2016 to December 2020
Asokkumar 2023	Singapore and Thailand	Retrospective cohort	November 2020 to August 2021
Badurdeen 2021	Brazil	Retrospective cohort	November 2017 to July 2018
Barrichello 2019	Brazil	Retrospective cohort	July 2017 to August 2018
Bhandari 2020	India	Retrospective cohort	March 2017 to October 2018
Brethauer 2012	USA	Prospective, multicenter, single‐arm feasibility cohort	N/A
Carr 2022	Australia	Prospective cohort	June 2018 to May 2019
Castagneto‐Gissey 2024	Italy	Retrospective cohort	January 2022 to December 2023
Cheskin 2020	USA	Retrospective case‐matched comparative cohort	2016 to 2018
Correia 2023	USA	Retrospective cohort	September 2021 to April 2022
Devière 2008	Belgium and Mexico	Prospective observational single‐arm (before/after) cohort	N/A
Espinet‐Coll 2022	Spain	Prospective cohort	From January 2017–(at least 1 year follow‐up)
Familiari 2011	Belgium and Italy	Prospective observational single‐arm (before/after) cohort	January 2007 to February 2009
Farha 2021	USA	Retrospective cohort	March 2016 to August 2019
Fayad 2019	USA	Retrospective cohort	December 2015 to October 2017
Fiorillo 2020	France	Retrospective single‐center cohort	October 2016 to July 2018
Galva Neto 2020	Brazil	Retrospective cohort	April 2017 to December 2018
Glaysher2019	UK	Retrospective cohort	January 2016 to December 2017
Hajifathalian 2021	USA	Prospective cohort	August 2013 to August 2019
Huberty 2018	Multi‐national	Retrospective cohort	November 2015 to December 2016
James 2020	USA	Retrospective cohort	May 2018 to July 2019
Kozlowska 2022	Poland	Retrospective cohort	2015 to 2020
Limas 2024	Indonesia	Retrospective cohort	June 2022 to June 2023
Lopez‐Nava 2021	Spain	Retrospective cohort	May 2013 to March 2020
Maselli 2023	USA	Retrospective cohort	May 2018 to March 2022
Matteo 2024	Italy	Retrospective cohort	May 2017 to March 2022
Nigro 2025	Italy	Prospective, randomized controlled, monocentric study (RCT)	N/A
Polese 2022	Italy	Retrospective cohort	January 2019 to March 2022
Sartoretto 2018	Australia and USA	Retrospective cohort	February 2016 and May 2017
Sharaiha 2021	USA	Prospective cohort	August 2013 to August 2019
Silva 2025	Colombia	Retrospective cohort	January 2022 to July 2023
Valentin Monaco 2025	North America	Retrospective cohort	2020 to 2023

**TABLE 2:** Patients' baseline characteristics.

Study ID	No. of patients	Age	Sex (Female/Male)	Baseline BMI	Comorbidities					
					Diabetes	Hypertension	Obstructive sleep apnea (OSA)	GERD	Hyperlipidemia/Dyslipidemia	Smoking
Abad 2025	20	55.15 ± 10.9	9/11	37.54 ± 4.81	9 (45)	11 (55)	N/A	N/A	12 (69)	N/A
Alqahtani 2022	3018	33.8 ± 9.6	2686/332	32.5 ± 3.1	112 (3.7)	101 (3.3)	N/A	N/A	62 (2.1)	N/A
Asokkumar 2023	18	42 ± 8.5	11/7	34.9 ± 4.4	4 (22)	7 (39)	7 (39)	N/A	7 (39)	N/A
Badurdeen 2021	ESG alone (26)	41.15 ± 10.64	16/10	35.56 ± 1.68	N/A	16 (61.5)	13 (50)	N/A	14 (53.8)	N/A
	ESG plus liraglutide (26)	40.65 ± 8.69	17/9	35.83 ± 2.33	N/A	10 (38.5)	13 (50)	N/A	12 (46.2)	N/A
Barrichello 2019	193	42.3 ± 9.6	148/45	34.11 ± 2.97	N/A	N/A	N/A	N/A	N/A	N/A
Bhandari 2020	53	40.54 ± 13.79	43/10	34.78 ± 5.2	10 (18.86)	15 (28.3)	6 (11.32)	4 (7.54)	N/A	N/A
Brethauer 2012	18	40^a^	N/A	38.6 ± 4.2	N/A	6 (42.9)	4 (28.6)	8 (57.1)	14 (100)	N/A
Carr 2022	16	41.4 ± 10.4	13/3	35.5 ± 5.2	0 (0)	7 (44)	0 (0)	4 (25)	3 (19)	N/A
Castagneto‐Gissey 2024	13	N/A	9/4	36.5 ± 3.9	N/A	N/A	N/A	N/A	N/A	N/A
Cheskin 2020	105	47.58 ± 11.97	75/30	40.5 ± 7.89	N/A	N/A	N/A	N/A	N/A	N/A
Correia 2023	73	49.19 ± 9.67	61/12	38.6 ± 3.51	26 (35.6)	28 (38.44)	10 (13.7)	18 (24.7)	19 (26)	17 (23.3)
Devière 2008	21	43.7 ± 9.7	17/4	43.3 ± 5	7 (33)	8 (40)	N/A	N/A	3 (15)	N/A
Espinet‐Coll 2022	38	47 [40.0–51.0]	30/8	N/A	N/A	N/A	N/A	N/A	N/A	N/A
Familiari 2011	67	41 ± 9.7	47/20	41.5 ± 3.6	14 (20.9)	27 (40)	N/A	N/A	23 (34)	N/A
Farha 2021	ESG‐FS [fundal suturing] (149)	47.16 ± 11.47	107/42	39.38 ± 7.33	12 (8.1)	37 (24.8)	N/A	N/A	N/A	N/A
	ESG‐NFS [without fundal suturing] (98)	44.94 ± 9.39	84/14	38.34 ± 5.56	4 (4.1)	29 (29.6)	N/A	N/A	N/A	N/A
Fayad 2019	58	48.2 ± 11.8	34/24	41.5 ± 8.2	3 (5.2)	17 (29.3)	9 (15.5)	11 (19)	N/A	N/A
Fiorillo 2020	23	41^a^	16/7	39.5^a^	2 (8.7)	3 (13)	5 (22)	N/A	N/A	N/A
Galvao Neto 2020	233	42.2	170/63	34.7	12	49	44	25	N/A	N/A
Glaysher2019	ESG + No longitudinal compression (9)	45 ± 12	5/4	35.9 (30.9–43.8)	N/A	N/A	N/A	N/A	N/A	N/A
	ESG + Longitudinal compression (23)	43 ± 10	18/5	36.5 (29.8–42.9)	N/A	N/A	N/A	N/A	N/A	N/A
Hajifathalian 2021	118	46 ± 13	80/38	40 ± 7	35	N/A	N/A	N/A	N/A	N/A
Huberty 2018	51	40.9 ± 10.3	45/6	35.1 ± 3	N/A	N/A	N/A	N/A	N/A	N/A
James 2020	100	45 ± 9	86/14	38.4 ± 5.4	4 (4)	29 (29)	N/A	N/A	13 (13)	N/A
Kozlowska 2022	42	43.8 ± 8.8	29/13	44.8 ± 4.5	N/A	N/A	N/A	N/A	N/A	N/A
Limas 2024	20	40.05 ± 10.54	17/3	31.12 ± 5.88	N/A	N/A	N/A	N/A	N/A	N/A
Lopez‐Nava 2021	435	48.9	314/121	39.4 ± 5.4	N/A	N/A	N/A	N/A	N/A	N/A
Maselli 2023	404	42.9 ± 9.4	317/87	44.8 ± 4.7	72 (17.8)	143 (35.39)	N/A	N/A	68 (16.83)	N/A
Matteo 2024	315	F 44 (35–54), M 49 (41–56)	230/85	F 36.1 (34.2–39.4), M 39.2 (36.0–43.7)	17 (5.39)	84 (26.67)	29 (9.2)	N/A	N/A	88 (27.93)
Nigro 2025	23	43.5 ± 7.9	21/2	34.4 ± 2.89	0 (0)	4 (17.39)	1 (4.34)	N/A	5 (21.73)	N/A
Polese 2022	27	N/A	N/A	36 ± 9	N/A	N/A	N/A	N/A	N/A	N/A
Sartoretto 2018	121	N/A	N/A	N/A	N/A	N/A	N/A	N/A	N/A	N/A
Sharaiha 2021	216	46 ± 13	146/70	39 ± 6	67	N/A	N/A	N/A	N/A	N/A
Silva 2025	90	42.2 ± 12.1	76/14	33 [29.4–36.8]	N/A	N/A	N/A	N/A	N/A	N/A
Valentin Monaco 2025	2285	45.1	1970/315	39.6	2000	704	N/A	627	329	N/A

*Note:* Continuous values are represented by Mean ± SD, median (range), or median [interquartile range]. Dichotomous values are represented by number (%).

Abbreviation: BMI, body mass index.

^a^ Mean only.

### 3.1 Quality Assessment

Fifteen studies yielded high quality, while sixteen were deemed moderate quality as determined by applying the NOS for cohort studies, as shown in Table 3. Furthermore, two studies were RCTs and were evaluated using the RoB2 tool; both were considered to have some concerns, as illustrated in Figure S1.

**TABLE 3:** Quality assessment of cohort studies using the Newcastle‐Ottawa scale.

Study ID	Newcastle‐Ottawa scale for cohort studies								Total score (out of 9)
	Selection (maximum: ★★★★)				Comparability (maximum: ★★)	Outcome (maximum: ★★★)			
	Representativeness of the exposed cohort (maximum: ★)	Selection of the non‐exposed cohort (maximum: ★)	Ascertainment of exposure (maximum: ★)	Demonstration that outcome of interest was not present at start of study (maximum: ★)	Comparability of cohorts on the basis of the design or analysis controlled for confounders	Assessment of outcome(maximum: ★)	Was follow‐up long enough for outcomes to occur (maximum: ★)	Adequacy of follow‐up of cohorts (maximum: ★)	
Alqahtani 2022	★	★	★	★	★★	★	★	★	9
Asokkumar 2023	★		★	★		★	★	★	6
Badurdeen 2021	★	★	★	★	★★	★	★	★	9
Barrichello 2019	★		★	★		★	★	★	6
Bhandari 2020	★		★	★		★	★	★	6
Brethauer 2012	★		★	★		★	★	★	6
Carr 2022	★	★	★	★		★	★		6
Castagneto‐Gissey 2024	★	★	★	★		★	★	★	7
Cheskin 2020	★	★	★	★	★	★	★		7
Correia 2023	★		★	★		★		★	5
Devière 2008	★		★	★		★	★	★	6
Espinet‐Coll 2022	★		★	★		★	★	★	6
Familiari 2011	★		★	★		★	★	★	6
Farha 2021	★	★	★	★	★★	★	★	★	9
Fayad 2019	★	★	★	★	★★	★	★	★	9
Fiorillo 2020	★	★	★	★	★	★	★	★	8
Galva Neto 2020	★	★	★	★	★★	★	★	★	9
Glaysher2019	★	★	★	★	★★	★	★	★	9
Hajifathalian 2021	★		★	★		★	★	★	6
Huberty 2018	★	★	★	★	★★	★	★	★	9
James 2020	★		★	★		★	★	★	6
Kozlowska 2022	★	★	★	★	★★	★	★	★	9
Limas 2024	★	★	★	★	★★	★	★	★	9
Lopez‐Nava 2021		★	★	★	★	★	★	★	7
Maselli 2023	★		★	★		★	★	★	6
Matteo 2024			★	★		★	★	★	5
Polese 2022	★		★	★		★	★	★	6
Sartoretto 2018		★	★	★	★	★	★	★	7
Sharaiha 2021	★		★	★		★	★		5
Silva 2025			★	★		★	★	★	5
Valentin Monaco 2025		★	★	★	★	★	★	★	7

### 3.2 Study Outcomes

#### 3.2.1 Short‐Term Outcomes

##### 3.2.1.1 Postoperative Short‐Term Complications

**FIGURE 3:**
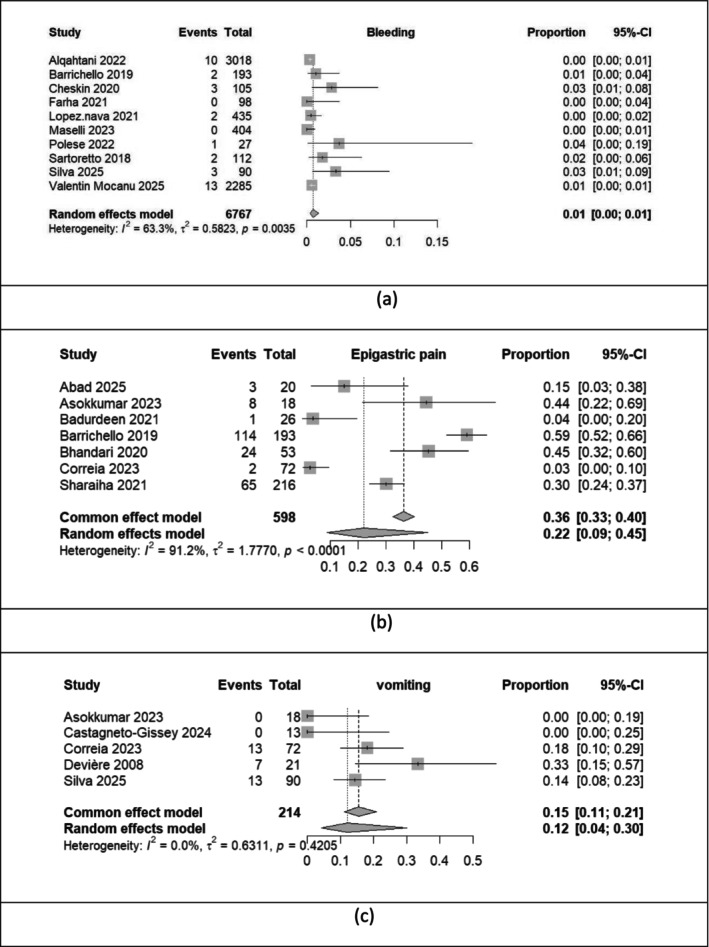
Post‐operative complications: (a) Bleeding, (b) Epigastric pain, and (c) Vomiting.

Analysis of postoperative complications, such as bleeding, epigastric pain, and vomiting, showed a pooled proportion of 1% (95% CI: 0.00–0.01; *I* ^2^ = 63.3%), 22% (95% CI: 0.09–0.45; *I* ^2^ = 91.2%), and 12% (95% CI: 0.04–0.30; *I* ^2^ = 0%), respectively, as illustrated in Figure 3. The high heterogeneity in epigastric pain may be attributed to variability in outcome definitions, pain assessment methods, and follow‐up durations across the studies. In contrast, heterogeneity for vomiting was absent, suggesting more consistent reporting of this outcome. Sensitivity analysis showed that exclusion of the study by Alqahtani et al. reduced heterogeneity for the bleeding outcome from 63.3% to 48.4%.

##### 3.2.1.2 Primary Continuous Outcomes

**FIGURE 4:**
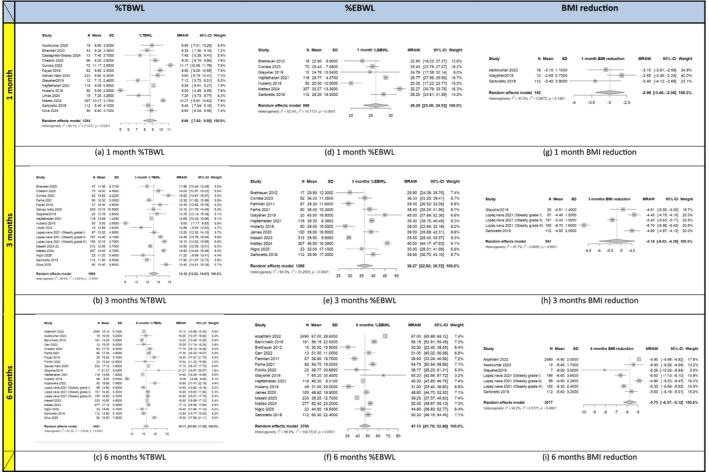
Short‐term primary outcomes.

Short‐term continuous outcomes were assessed into three different subgroups: 1, 3, and 6 months of follow‐up periods. Although high heterogeneity was observed across studies, %TBWL and %EBWL demonstrated consistent progressive improvement over time. The pooled means for %TBWL at 1, 3, and 6 months were 8.66% (*I* ^2^ = 96.1%), 13.12% (*I* ^2^ = 95%), and 16.13% (*I* ^2^ = 93.3%), respectively (Figure 4a–c). Furthermore, the pooled means for %EBWL were 26.29% (*I* ^2^ = 92.6%), 36.27% (*I* ^2^ = 96.8%), and 47.13% (*I* ^2^ = 98.8%), respectively (Figure 4d–f). In addition, the pooled mean BMI reduction was 2.98 kg/m^2^ (*I* ^2^ = 47.6%), 5.19 kg/m^2^ (*I* ^2^ = 97.7%), and 5.75 kg/m^2^ (*I* ^2^ = 94.2%), respectively. Sensitivity analysis demonstrated that exclusion of the study by Glaysher et al. reduced heterogeneity for the 1‐month BMI reduction outcome from 47.6% to 0%.

#### 3.2.2 Long‐Term Outcomes

##### 3.2.2.1 Primary Continuous Outcomes

**FIGURE 5:**
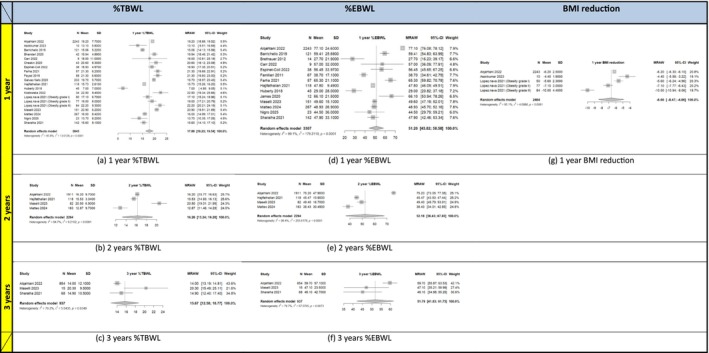
Long‐term primary outcomes.

The outcomes for the long‐term duration were assessed into three subgroups: 1, 2, and 3 years of follow‐up. In contrast to the short‐term outcomes, %TBWL demonstrated a gradual decline over time, although substantial heterogeneity remained across studies. Pooled means for %TBWL were 17.88% (*I* ^2^ = 95.8%), 16.26% (*I* ^2^ = 94.7%), and 15.67% (*I* ^2^ = 70.2%), respectively (Figure 5a–c). Sensitivity analysis for the 3‐year %TBWL outcome demonstrated a reduction in heterogeneity from 70.2% to 0% following exclusion of the study by Maselli et al.

Moreover, the pooled mean for %EBWL was 51.2% (*I* ^2^ = 99.1%), 52.18% (*I* ^2^ = 99.4%), and 51.79% (*I* ^2^ = 79.7%), respectively (Figure 5d–f). Sensitivity analysis demonstrated that exclusion of the study Alqahtani 2022 reduced heterogeneity for the 1 year %EBWL outcome from 99.1% to 90.8%, and for the 3‐year %EBWL outcome from 79.7% to 0%.

Furthermore, the BMI reduction pooled mean was reduced at 1 year follow‐up by 6.66 kg/m^2^ (*I* ^2^ = 95.1%), while no pooled analyses were available for 2 or 3 year BMI reduction outcomes due to insufficient reporting across studies (Figure 5g); sensitivity analysis showed a drop of heterogeneity from 95.1% to 84.4% by excluding the obesity grade III patients from the study by Lopez. Nava et al.

##### 3.2.2.2 Remission Rates of Comorbidities

**FIGURE 6:**
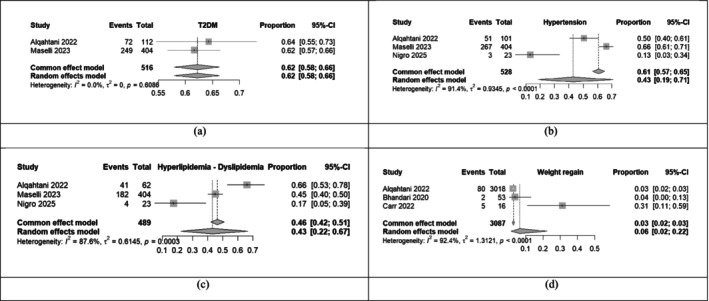
Long‐term remission rates of (a) Type 2 Diabetes Mellitus, (b) Hypertension, and (c) Hyperlipidaemia/Dyslipidaemia; and (d) Long‐term weight re‐gain.

Type 2 diabetes mellitus showed the highest remission rate, with a pooled proportion of 62% (95% CI: 0.58–0.66; *I* ^2^ = 0%), while the remission rate for hypertension was 43% (95% CI: 0.19–0.71; *I* ^2^ = 91.4%), and that for dyslipidaemia was 43% (95% CI: 0.22–0.67; *I* ^2^ = 87.6%), as presented in Figure 6a–c.

##### 3.2.2.3 Weight Regain

Three studies assessed long‐term weight regain (defined as any increase in body weight after reaching nadir weight or regain of > 20% of the initially lost weight during follow‐up periods of approximately 4–12 months) [26, 30, 32], yielding a pooled proportion of 6% (95% CI: 0.02–0.22; *I* ^2^ = 92.4%), as shown in Figure 6d.

#### 3.2.3 Short‐Term Versus Long‐Term Outcomes

**FIGURE 7:**
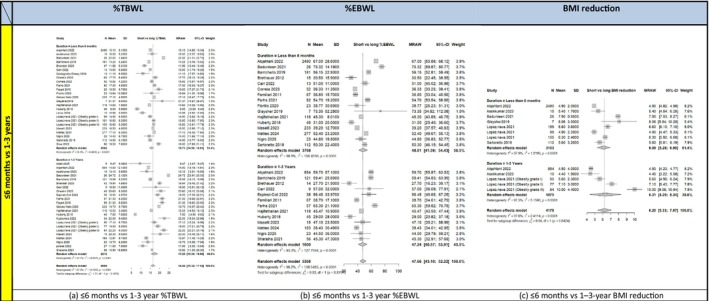
≤ 6 months vs. 1–3‐year primary outcomes.

Short‐term outcomes were defined as the longest reported follow‐up within 1–6 months, whereas long‐term outcomes were defined as the longest reported follow‐up within 1–3 years after the procedure. %TBWL, %EBWL, and BMI reduction showed a consistent increase from short‐term to long‐term follow‐up, while high heterogeneity remained evident across studies. The pooled mean for %TBWL in the short‐ and long‐term follow‐up was 15.71% (*I* ^2^ = 96.3%) and 17.2% (*I* ^2^ = 97.1%), respectively; subgroup analysis showed no statistically significant difference between short and long‐term %TBWL outcomes, suggesting comparable weight loss across follow‐up durations (Figure 7a, *p* = 0.1876). The pooled mean for %EBWL was 48.01% (*I* ^2^ = 98.9%) and 47.24% (*I* ^2^ = 93.3%), respectively; subgroup analysis was statistically insignificant (Figure 7b, *p* = 0.8701). Furthermore, the pooled mean for BMI reduction showed a decrease of 6.09 kg/m^2^ (*I* ^2^ = 97.9%) and 6.31 kg/m^2^ (*I* ^2^ = 97.5%), respectively; subgroup analysis was statistically insignificant (Figure 7c, *p* = 0.8424).

**FIGURE 8:**
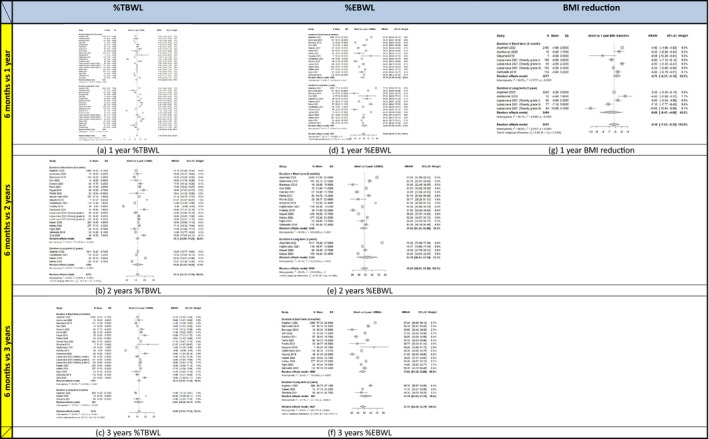
Short‐term versus long‐term primary outcomes.

An additional analysis was performed using studies that reported a 6‐month follow‐up as the short‐term reference point, which was subsequently compared with 1, 2, and 3‐year follow‐up outcomes to further evaluate the differences between short‐ and long‐term follow‐up results. %TBWL comparison between 6 months follow‐up and 1, 2, and 3 years follow‐up showed statistically insignificant subgroup differences with *p* values of 0.86, 0.9365, and 0.7862 (Figure 8a–c). Similarly, %EBWL subgroup differences did not reach statistical significance with *p* values of 0.3816, 0.5466, and 0.4171 (Figure 8d–f). BMI reduction comparison at 1‐year follow‐up showed an insignificant subgroup *p* value of 0.3466 (Figure 8g). However, due to overlapping cohorts across multiple time points, these comparisons should be interpreted cautiously as descriptive trajectories rather than definitive tests of durability over time.

A summary of the secondary outcomes is presented in Table 4, with the corresponding forest plots shown in Figure S5. While re‐admission, re‐operation, and mortality rates were reported, the time frame within which these outcomes occurred was not consistently defined across the included studies.

**TABLE 4:** Summary of the secondary outcomes.

Outcome	Figure number	Number of studies	Effect estimate	Effect size (%95 CI)	Heterogeneity (*I* ^2^)
LOHS	Figure S3a	5	Mean difference	1.31 (0.57–2.05)	99.6%
Readmission rate	Figure S3b	4	Pooled proportion	4% (0.02–0.08)	67.6%
Reoperation rate	Figure S3c	4	Pooled proportion	2% (0–0.08)	90.4%
Mortality rate	Figure S3d	4	Pooled proportion	0% [N/A]	N/A

### 3.3 Publication Bias

Funnel plot assessments and Egger's regression tests were conducted for continuous primary outcomes (%TBWL, %EBWL, and BMI reduction) across short‐term, long‐term, and comparative short‐term versus long‐term analyses. Visual inspection revealed funnel plot asymmetry across all assessed outcomes except postoperative bleeding, 3‐months %TBWL, 3‐months %EBWL, and 1‐year %EBWL outcomes. While Egger's test did not reach statistical significance except for 6 months BMI reduction and 1‐year %EBWL outcomes, funnel plot asymmetry in several analyses may suggest small‐study effects and heterogeneity. Funnel plots can be seen in the Figures S2–S4.

### 3.4 Meta Regression Analysis

To explore potential sources of heterogeneity and assess the influence of patient baseline BMI on weight loss outcomes following the procedure, we performed meta‐regression analyses for each continuous primary outcome.

Higher baseline BMI was a significant predictor of greater %TBWL in both short‐ and long‐term follow‐ups (estimate = 0.69, *p* < 0.0001) and (estimate = 0.48, *p* = 0.001) respectively. Conversely, higher BMI was significantly associated with lower %EBWL in the short‐term (estimate = −1.5, *p* = 0.03) and long‐term (estimate = −1.89, *p* = 0.006). Higher baseline BMI was also associated with a significantly lower reduction in BMI change at long‐term follow‐up (estimate = −0.34, *p* = 0.02); the short‐term relationship was not significant (*p* = 0.25). Baseline BMI explained between 8.6% and 24.3% of the between‐study heterogeneity across the various outcome models, though substantial unexplained variability remained (*I* ^2^ > 97%). A visual representation of the meta‐regression is seen in bubble plots in Figure S9.

### 3.5 GRADE Assessment

As per the GRADE assessment, primary continuous outcomes and remission of comorbidities were evaluated. The certainty of evidence for all outcomes ranged from moderate to very low. Certainty was downgraded mainly for inconsistency (heterogeneity), imprecision (wide confidence intervals), and suspected publication bias where Egger's test was significant. Detailed GRADE ratings are provided in Table 5.

**TABLE 5:** GRADE assessment for primary continuous outcomes and comorbidities remission rates.

Outcome	No. of studies	Study design	Risk of bias	Inconsistency	Indirectness	Imprecision	Publication bias	Certainty of evidence
Short‐term %TBWL	Multiple	Observational + RCTs	Not serious	Serious	Not serious	Not serious	Undetected	Low
Short‐term %EBWL	Multiple	Observational + RCTs	Not serious	Serious	Not serious	Not serious	Undetected	Low
Short‐term BMI reduction	Multiple	Observational + RCTs	Not serious	Serious	Not serious	Not serious	Suspected	Low
Post‐operative bleeding	Multiple	Observational	Not serious	Moderate	Not serious	Not serious	Undetected	Moderate
Post‐operative epigastric pain	Multiple	Observational	Not serious	Serious	Not serious	Serious	Undetected	Low
Post‐operative vomiting	Multiple	Observational	Not serious	Serious	Not serious	Serious	Undetected	Low
Long‐term %TBWL	Multiple	Observational + RCTs	Not serious	Serious	Not serious	Not serious	Undetected	Low
Long‐term %EBWL	Multiple	Observational + RCTs	Not serious	Serious	Not serious	Moderate	Suspected	Low
Long‐term BMI reduction	Multiple	Observational	Not serious	Serious	Not serious	Moderate	Undetected	Low
Type 2 diabetes remission	Multiple	Observational	Not serious	Not serious	Not serious	Not serious	Undetected	Moderate
Hypertension remission	Multiple	Observational	Not serious	Serious	Not serious	Moderate	Undetected	Low
Dyslipidaemia remission	Multiple	Observational	Not serious	Serious	Not serious	Moderate	Undetected	Low
Weight regain	4	Observational	Serious	Serious	Not serious	Serious	Undetected	Very low
Short‐ vs. long‐term %TBWL	Multiple	Observational + RCTs	Not serious	Serious	Not serious	Not serious	Undetected	Low
Short‐ vs. long‐term %EBWL	Multiple	Observational + RCTs	Not serious	Serious	Not serious	Not serious	Undetected	Low
Short‐ vs. long‐term BMI reduction	Multiple	Observational + RCTs	Not serious	Serious	Not serious	Not serious	Undetected	Low

## 4 Discussion

In this systematic review and meta‐analysis, we evaluated both the short‐term and longer‐term safety and efficacy profiles of endoscopic sleeve gastroplasty (ESG). In our study, we synthesized evidence by incorporating 33 studies, pooling 8880 individuals. This provides one of the largest assessments of the performance of ESG beyond the scope of the earlier, shorter‐term reviews [11, 12, 56].

ESG was associated with clinically meaningful weight loss that appears generally sustained beyond the early post‐procedural period. Short‐term analysis showed progressive increases in the %TBWL and %EBWL during the first 6 months. This aligns with the previously published meta‐analyses [11, 12, 56]. On longer‐term follow‐up, the pooled %TBWL and %EBWL remained clinically relevant. Comparisons across different follow‐up periods highlighted greater pooled weight reduction estimates at later time points. This suggests that the weight loss effects of ESG can extend beyond the early post‐procedural period.

However, these findings must be interpreted with caution due to the high heterogeneity observed across most major outcomes. We explored potential sources of this heterogeneity through sensitivity analyses, meta‐regression, and qualitative synthesis of the included studies. Between‐study variability may arise from multiple confounding factors. Differences in centre experience, procedural volume, and procedural techniques (e.g., suturing techniques and fundal involvement) may contribute to the observed heterogeneity. Differences in baseline patient characteristics (particularly BMI) and the use of adjunct pharmacotherapy may also act as contributing factors.

Different study designs, inconsistent outcome definitions, and follow‐up periods may be playing a significant role in limiting the precision of the pooled outcomes. While meta‐regression demonstrated baseline BMI as a significant predictor of %TBWL and %EBWL, considerable unexplained variability remained. This is consistent with the previously demonstrated challenges in creating evidence for endoscopic bariatric procedures and chronic metabolic disease management [57, 58].

Despite the high heterogeneity, leave‐one‐out sensitivity analyses demonstrated that no single study disproportionately altered the overall effect. Comparative analyses of short‐ versus long‐term outcomes revealed no major differences. As such, the direction of weight reduction and the resulting metabolic effects remained relatively stable over time.

Improvements in metabolic outcomes highlight the clinical benefits of ESG. Remission of type 2 diabetes mellitus was most pronounced. That was followed by hypertension and dyslipidaemia. While heterogeneity was high, the direction of effect was consistently positive. Weight regain was infrequently reported and occurred in only a small proportion of patients. Again, this must be interpreted cautiously due to inconsistent definitions across studies and variable longer‐term follow‐up [59]. These findings align with the growing evidence that certain interventions can modify metabolic disease burden over time [60].

In terms of safety, serious adverse events were rare and consistent with previously published data [11, 12]. More common symptoms, such as epigastric pain and vomiting, were transient. Secondary outcomes, such as re‐operations, re‐admissions, and mortality, also occurred at low rates. However, the variability in reporting timeframes for secondary outcomes adds further uncertainty.

The findings of this meta‐analysis suggest that ESG may present a practical, minimally invasive option within metabolic obesity management. It appears to provide meaningful weight reduction with a generally favourable safety profile in the studied populations. That is relevant to patients who are unsuitable for or unwilling to undergo bariatric surgery. In selected cases, it could potentially act as a relatively safer bridging procedure before undergoing a more complex procedure. The identification of baseline BMI as a factor affecting weight reduction highlights the importance of patient selection and individualizes the expected outcomes. Additionally, the observed metabolic improvements, especially in DM, support the ESG as a metabolic disease intervention rather than simply being a weight‐reducing procedure.

Our study has several strengths that enhance the reliability of the findings. It represents one of the largest and most comprehensive analyses of ESG outcomes to date. It also presents the longer‐term follow‐ups and stratifies outcomes by time intervals. In addition, sensitivity analyses demonstrate that the overall direction of the pooled results remained consistent.

Several limitations in our study should be acknowledged. Most of the included studies were observational studies and single‐arm in nature. Definitions of outcomes, especially weight regain, varied across the included studies. This constrained the precise pooling and contributed to the high heterogeneity. High heterogeneity was observed across many of the pooled outcomes. This may be attributed to the variability in study design, patient selection, baseline BMI, procedural technique, follow‐up durations, and reporting practice. These factors could not be fully explained through meta‐regression. This results in reduced certainty of the pooled estimates. Additionally, they highlight the need for greater standardization in future research. Finally, the inclusion of repeated data points across different follow‐up intervals from the same cohorts limits the independence of some comparisons.

In conclusion, endoscopic sleeve gastroplasty appears to be a relatively safe and effective intervention that may achieve clinically acceptable weight reduction. It also demonstrates improved metabolic outcomes beyond the early follow‐up periods. By synthesizing such a large and heterogeneous body of real‐world evidence, this study provides an estimate of the safety and efficacy profile of ESG in the field of metabolic surgery. However, due to the high heterogeneity driven by multiple confounding factors, these findings should be considered within individualized multidisciplinary care rather than definitive evidence. This emphasizes the need for longer‐term high‐quality studies for better characterization of long‐term outcomes.

## Author Contributions

A.A. and M.B. created the research idea. A.A., M.R., A.E., A.E., K.M., O.A., M.A., M.I.E., and Z.M.W. have all participated in designing the research workflow, acquisition and interpretation of data, and screening of the data. M.B., A.A., M.R., Y.K., A.E., and K.M. have participated in the writing, editing, and reviewing of the manuscript. A.A., M.B., M.R., Y.K., and K.M. have analysed the results. M.R. performed the statistical analysis and created the figures. M.B., A.A., M.R., A.E., A.E., K.M., T.S., and H.T. have revised the whole work. All authors have read and agreed to the final version of the manuscript.

## Funding

The authors have nothing to report. Funding sources for the 33 included studies are summarized in Table S3.

## Ethics Statement

Ethical (IRB) approval for the 33 included studies is summarized in Table S3.

## Consent

The authors have nothing to report.

## Conflicts of Interest

The authors declare no conflicts of interest.

## Supporting information


**Data S1:** PRISMA 2020 Checklist.
**Table S1:** PRISMA‐2020 checklist.
**Table S2:** DOI table.
**Table S3:** Ethical approval and funding source for each study.
**Figure S1:** Risk of bias assessment of randomized controlled trials using the RoB 2 tool.
**Figure S2:** Funnel plots of post‐operative complications.
**Figure S3:** Short‐term continuous outcomes funnel plots.
**Figure S4:** Long‐term continuous outcomes funnel plots.
**Figure S5:** Post‐operative secondary outcomes.
**Figure S6:** Leave‐one‐out sensitivity analyses of post‐operative complications: (a) Bleeding, (b) Epigastric pain, and (c) Vomiting.
**Figure S7:** Leave‐one‐out sensitivity analyses of continuous primary short‐term outcomes.
**Figure S8:** Leave‐one‐out sensitivity analyses of continuous primary long‐term outcomes.
**Figure S9:** Meta‐regression bubble plots of continuous primary outcomes.

## Data Availability

All data analysed during this study are included in this published article and its Supporting Information files.
