# Supplementary material for: Short‐Term Versus Long‐Term Efficacy of Endoscopic Sleeve Gastroplasty in 8880 Obese Patients: A Systematic Review and Meta‐Analysis
Source: Diabetes Obes Metab. 2026 Jun 18;28(9):8189–207. doi: 10.1111/dom.70988 (PMC13449078; doi:10.1111/dom.70988)
Supplement: Supplementary file 1 [file DOM-28-8189-s001.docx]

**PRISMA 2020 Checklist**

| **Section and Topic** | **Item #** | **Checklist item** | **Location where item is reported** |
| --- | --- | --- | --- |
| **TITLE** | | |  |
| **Title** | **1** | **Identify the report as a systematic review.** | Page 1 |
| **ABSTRACT** | | |  |
| **Abstract** | **2** | **See the PRISMA 2020 for Abstracts checklist.** | Page 2 |
| **INTRODUCTION** | | |  |
| **Rationale** | **3** | **Describe the rationale for the review in the context of existing knowledge.** | Page 2 |
| **Objectives** | **4** | **Provide an explicit statement of the objective(s) or question(s) the review addresses.** | Page 3 |
| **METHODS** | | |  |
| **Eligibility criteria** | **5** | **Specify the inclusion and exclusion criteria for the review and how studies were grouped for the syntheses.** | Page 3 |
| **Information sources** | **6** | **Specify all databases, registers, websites, organisations, reference lists and other sources searched or consulted to identify studies. Specify the date when each source was last searched or consulted.** | Page 3 |
| **Search strategy** | **7** | **Present the full search strategies for all databases, registers and websites, including any filters and limits used.** | Page 3 |
| **Selection process** | **8** | **Specify the methods used to decide whether a study met the inclusion criteria of the review, including how many reviewers screened each record and each report retrieved, whether they worked independently, and if applicable, details of automation tools used in the process.** | Page 4 |
| **Data collection process** | **9** | **Specify the methods used to collect data from reports, including how many reviewers collected data from each report, whether they worked independently, any processes for obtaining or confirming data from study investigators, and if applicable, details of automation tools used in the process.** | Page 4 |
| **Data items** | **10a** | **List and define all outcomes for which data were sought. Specify whether all results that were compatible with each outcome domain in each study were sought (e.g. for all measures, time points, analyses), and if not, the methods used to decide which results to collect.** | Page 4 |
|  | **10b** | **List and define all other variables for which data were sought (e.g. participant and intervention characteristics, funding sources). Describe any assumptions made about any missing or unclear information.** | Page 4 |
| **Study risk of bias assessment** | **11** | **Specify the methods used to assess risk of bias in the included studies, including details of the tool(s) used, how many reviewers assessed each study and whether they worked independently, and if applicable, details of automation tools used in the process.** | Page 4 |
| **Effect measures** | **12** | **Specify for each outcome the effect measure(s) (e.g. risk ratio, mean difference) used in the synthesis or presentation of results.** | Page 4 |
| **Synthesis methods** | **13a** | **Describe the processes used to decide which studies were eligible for each synthesis (e.g. tabulating the study intervention characteristics and comparing against the planned groups for each synthesis (item #5)).** | Page 4 |
|  | **13b** | **Describe any methods required to prepare the data for presentation or synthesis, such as handling of missing summary statistics, or data conversions.** | Page 4 |
|  | **13c** | **Describe any methods used to tabulate or visually display results of individual studies and syntheses.** | Page 5 |
|  | **13d** | **Describe any methods used to synthesize results and provide a rationale for the choice(s). If meta-analysis was performed, describe the model(s), method(s) to identify the presence and extent of statistical heterogeneity, and software package(s) used.** | Page 4 |
|  | **13e** | **Describe any methods used to explore possible causes of heterogeneity among study results (e.g. subgroup analysis, meta-regression).** | Page 5 |
|  | **13f** | **Describe any sensitivity analyses conducted to assess robustness of the synthesized results.** | Page 5 |
| **Reporting bias assessment** | **14** | **Describe any methods used to assess risk of bias due to missing results in a synthesis (arising from reporting biases).** | Page 5 |
| **Certainty assessment** | **15** | **Describe any methods used to assess certainty (or confidence) in the body of evidence for an outcome.** | Page 4 |
| **RESULTS** | | |  |
| **Study selection** | **16a** | **Describe the results of the search and selection process, from the number of records identified in the search to the number of studies included in the review, ideally using a flow diagram.** | Page 5 |
|  | **16b** | **Cite studies that might appear to meet the inclusion criteria, but which were excluded, and explain why they were excluded.** | - |
| **Study characteristics** | **17** | **Cite each included study and present its characteristics.** | Page 5 |
| **Risk of bias in studies** | **18** | **Present assessments of risk of bias for each included study.** | Page 5 |
| **Results of individual studies** | **19** | **For all outcomes, present, for each study: (a) summary statistics for each group (where appropriate) and (b) an effect estimate and its precision (e.g. confidence/credible interval), ideally using structured tables or plots.** | Page 5 |
| **Results of syntheses** | **20a** | **For each synthesis, briefly summarise the characteristics and risk of bias among contributing studies.** | Page 6-7 |
|  | **20b** | **Present results of all statistical syntheses conducted. If meta-analysis was done, present for each the summary estimate and its precision (e.g. confidence/credible interval) and measures of statistical heterogeneity. If comparing groups, describe the direction of the effect.** | Page 6-7 |
|  | **20c** | **Present results of all investigations of possible causes of heterogeneity among study results.** | Page 7 |
|  | **20d** | **Present results of all sensitivity analyses conducted to assess the robustness of the synthesized results.** | Page 5&7 |
| **Reporting biases** | **21** | **Present assessments of risk of bias due to missing results (arising from reporting biases) for each synthesis assessed.** | Page 5&7 |
| **Certainty of evidence** | **22** | **Present assessments of certainty (or confidence) in the body of evidence for each outcome assessed.** | Page 7 |
| **DISCUSSION** | | |  |
| **Discussion** | **23a** | **Provide a general interpretation of the results in the context of other evidence.** | Page 8 |
|  | **23b** | **Discuss any limitations of the evidence included in the review.** | Page 9 |
|  | **23c** | **Discuss any limitations of the review processes used.** | page 9 |
|  | **23d** | **Discuss implications of the results for practice, policy, and future research.** | page 9 |
| **OTHER INFORMATION** | | |  |
| **Registration and protocol** | **24a** | **Provide registration information for the review, including register name and registration number, or state that the review was not registered.** | Page 3 |
|  | **24b** | **Indicate where the review protocol can be accessed, or state that a protocol was not prepared.** | page 3 |
|  | **24c** | **Describe and explain any amendments to information provided at registration or in the protocol.** | - |
| **Support** | **25** | **Describe sources of financial or non-financial support for the review, and the role of the funders or sponsors in the review.** | Page 10 |
| **Competing interests** | **26** | **Declare any competing interests of review authors.** | Page 10 |
| **Availability of data, code and other materials** | **27** | **Report which of the following are publicly available and where they can be found: template data collection forms; data extracted from included studies; data used for all analyses; analytic code; any other materials used in the review.** | Page 10 |

**Supplementary Table 1.** PRISMA-2020 checklist.

| **Study ID** | **DOI** | **No. of patients** | **Methodology summary** |
| --- | --- | --- | --- |
| Abad 2025 | 10.1016/j.cgh.2024.10.027 | 40 | Forty patients with biopsy-proven MASH (NAS ≥3, fibrosis F0–F3) were randomized to ESG plus lifestyle modification or sham endoscopy plus lifestyle intervention. Over 72 weeks, changes in weight, liver tests, liver stiffness, and histology were assessed among patients completing follow-up. |
| Alqahtani 2022 | 10.1016/j.gie.2022.02.050 | 3,018 | This was a propensity score–matched study of patients who underwent ESG or LSG. The primary outcome was weight loss at 6, 12, 24, and 36 months. A noninferiority margin of 10% total weight loss (%TWL) was used. Secondary outcomes were safety and comorbidity resolution. |
| Asokkumar 2023 | 10.1002/deo2.213 | 18 | We reviewed the records of 18 patients who underwent ESG using the novel device at the Singapore General Hospital, Singapore, and Siriraj Hospital, Bangkok, between 2020 and 2021. We adopted a "U" suture pattern. Our primary outcome was to assess technical feasibility and safety. The secondary outcome was to determine the percentage of total body weight loss at 1 year. |
| Badurdeen 2021 | 10.1016/j.gie.2020.10.016 | 52 | This retrospective study analyzed prospectively collected data from ESG patients at three outpatient clinics in Brazil (2017–2018). Patients who initiated liraglutide 5 months post-ESG were matched 1:1 to controls, with weight loss and body fat outcomes assessed 12 months after ESG. |
| Barrichello 2019 | 10.1016/j.gie.2019.06.013 | 193 | This multicenter study analyzed prospectively collected data from seven centers of patients with overweight or obesity undergoing ESG. Weight loss outcomes, body composition, metabolic rate, and adverse events were assessed at 6 and 12 months, with clinical success defined by ASGE/ASMBS thresholds. |
| Bhandari 2020 | 10.1111/den.13508 | 53 | We conducted a single-center retrospective study of obese patients who underwent consecutive ESG at our tertiary care center. Data on weight loss and adverse events at 1, 3, 6, and 12 months were collected and analyzed. |
| Brethauer 2012 | 10.1016/j.soard.2011.10.016 | 18 | Patients with a body mass index (BMI) of 30 – 45 kg/m2 were enrolled in the present institutional review board-approved study. Anterior to posterior gastric plications were placed in the gastric fundus and body using the suturing device. The endpoints were procedure time, adverse events, weight loss, and endoscopic findings at 1, 6, and 12 months after the procedure. The nominal P values are presented. |
| Carr 2022 | 10.1186/s12875-022-01629-7 | 61 | This two-arm prospective cohort study compared ESG and LSG in adults with obesity receiving multidisciplinary support, with follow-up to 12 months. The primary outcome was percent excess weight loss, with secondary outcomes including body composition, quality of life, metabolic markers, and adverse events. |
| Castagneto-Gissey 2024 | 10.20517/2574-1225.2024.31 | 13 | A single-center retrospective study was designed to evaluate the incidence of post-procedure symptoms in patients undergoing IGB and ESG. Incidence and severity of gastric symptoms were assessed using visual analog scales at various time points. Weight outcomes and medication usage were also recorded. Changes at different time points (baseline, one, and four months) were compared by means of the Mann-Whitney U Test. Bivariate correlations were carried out through Pearson correlation. |
| Cheskin 2020 | 10.1016/j.gie.2019.09.029 | 386 | This case-matched study compared ESG plus low-intensity diet and lifestyle therapy with high-intensity diet and lifestyle therapy alone, matching patients by age, sex, and BMI. Percent total body weight loss was assessed at 1, 3, 6, and 12 months. |
| Correia 2023 | 10.1007/s00464-023-09982-z | 73 | Patients with BMI 31.1–46.6 kg/m² underwent ESG, with efficacy and safety assessed at 1 and 3 months via excess and total weight loss. Statistical analyses included chi-square, paired t-tests, ANOVA, and Pearson’s correlation to explore associations with baseline factors. |
| Devière 2008 | 10.1007/s00464-007-9662-5 | 21 | The protocol was approved by the institutional review boards (IRBs) of both centers involved, and all patients gave informed consent. Patients met established inclusion criteria for bariatric surgery. The TOGa system (Satiety Inc., Palo Alto, CA), a set of transoral endoscopically guided staplers, was used to create a stapled restrictive pouch along the lesser curve of the stomach. Patients were hospitalized overnight for observation and underwent a barium upper gastrointestinal (UGI) the next morning. Post procedure, all patients were placed on a liquid diet for 1 month and asked to begin an exercise program. Follow-up was carried out at 1 week and 1, 3, 4, 5, and 6 months. |
| Espinet-Coll 2022 | 10.1007/s11695-022-06039-3 | 38 | This is a prospective, single-center study, including patients with obesity undergoing ESG with at least 1-year follow-up, who underwent the following: (1) Barium X-ray study (BS) prior and at 6 months, assessing degree of gastric reduction (severe, moderate, or mild), and (2) gastroscopy at 12 months, accounting the number of persistent sutures (tense, lax, or absent). Secondary outcomes were weight loss data and procedure safety profile. |
| Familiari 2011 | 10.1016/j.gie.2011.08.046 | 67 | Main inclusion criteria to the study were: BMI ranging between 40 and 55 kg/m2 (35 in the presence of obesity-related co- morbidities), no hiatal hernia, conformity with the standard physical, surgical, nutritional, and psychological criteria for BS. TOGa procedures were performed with two transoral prototype staplers, which were used to create a restrictive pouch along the lesser gastric curvature (volume about 30ml). Dietetic restrictions and exercises were strictly recommended after TOGa. The regular follow-up included physical examination, EGD, barium UGI, blood tests, and Quality-Of-Life evaluation. Only the patients who completed a 12-month follow-up were included in this report. |
| Farha 2021 | 10.1055/a-1236-9347 | 247 | We conducted a two-center retrospective analysis of 247 patients who underwent ESG with or without FS. The primary outcome was percent excess weight loss (%EWL) at 3, 6, and 12-months post-ESG. The secondary outcomes include the SAE rate and procedure duration. |
| Fayad 2019 | 10.1055/a-0852-3441 | 47 | This was a retrospective review of prospectively collected data from consecutive patients between December 2015 and October 2017 who underwent IGB or ESG at a single academic center. Fluid-filled IGBs implanted for a 6- month duration were used. IGB and ESG patients were subjected to identical post-procedure dietary instructions and follow-up protocols. Body weight was recorded at 1, 3, 6, and 12 months post-procedure. |
| Fiorillo 2020 | 10.1007/s11695-020-04419-1 | 23 | QoL was evaluated by means of the Gastrointestinal Quality of Life Index (GIQLI) questionnaire before and 6 months after the procedure. Patients were matched for age, sex, preoperative weight, and comorbidities. |
| Galvo Neto 2020 | https://doi.org/10.1007/s00464-019-07212-z | 233 | Patients undergoing ESG at four bariatric centers in Brazil between April 1, 2017 and December 31, 2018 were  prospectively enrolled in the study (BMI 30.0–39.9 kg/m2). ESG was performed using Overstitch (Apollo Endosurgery,  Austin, TX). Descriptive analysis, t test, Chi-square test, and Mann–Whitney test were used to present the results. |
| Glaysher2019 | 10.1055/a-0996-8089 | 32 | Retrospective review of prospectively collected data from consecutive adults undergoing endoscopic sleeve gastroplasty by a single operator at one UK center (2016–2017). Outcomes at up to 6 months were compared between a conventional triangular suture pattern and a modified longitudinal compression pattern, with weight-loss metrics and adverse events analyzed using appropriate parametric and non-parametric statistics. |
| Hajifathalian 2021 | 10.1016/j.gie.2020.08.023 | 118 | One hundred eighteen patients with obesity and NAFLD underwent ESG and were followed for 2 years. Weight loss was evaluated as % total body weight loss. IR was evaluated using the homeostasis model assessment of insulin resistatotalreviously validated hepatic steatosis index and NAFLD fibrosis score were used to estimate hepatic steatosis and risk of fibrosis. |
| Huberty 2018 | 10.1055/a-0630-1224 | 51 | After a pre-bariatric multidisciplinary work-up, class 1 and 2 obese patients were included. Using a simple triangulation platform, transmural sutures with serosa-to-serosa apposition were performed in the gastric cavity. Patients were followed according to the same routines as those performed for bariatric procedures. |
| James 2020 | 10.1007/s11695-019-04331-3 | 100 | One hundred patients underwent ESG (86 women, mean age 45 ± 9 years) and were analyzed for TBWL.. |
| Kozlowska 2022 | 10.1007/s11695-022-06398-x | 42 | We retrospectively analyzed the weight loss at IGB (Orbera) removal after 6 months (124 patients), at IGB (Orbera365) removal after 12 months (61 patients), and at 6 and 12 months after ESG (42 and 34 patients, respectively). Postprocedural care, including medication and diet, was the same for all procedures. |
| Limas 2024 | 10.7759/cureus.67355 | 20 | We retrospectively reviewed prospectively collected data for patients undergoing ESG or IGB. Weight was recorded at one week, one month, and three months post-procedure, and the percentage of total body weight loss (%TBWL) was calculated. |
| Lopez-Nava 2021 | https://doi.org/10.1007/s00464-021-08289-1 | 435 | We reviewed 484 patient records and identified 435 patients (class I: 105, class II: 169, class III: 161) who underwent ESG at our unit between May 2013 and March 2020. We compared their total body weight loss (%TBWL) and safetyover 1 year. We used a linear mixed model (LMM) to analyse repeated measures of weight loss outcomes at 3, 6, 9, and 12 months for comparison between the three BMI groups. |
| Maselli 2023 | 10.4253/wjge.v15.i6.469 | 404 | This was a retrospective cohort study that used prospectively collected data on adults with BMI ≥ 40 kg/m2 who underwent ESG and longitudinal lifestyle counseling at two centers with expertise in endobariatric therapies from May 2018 to March 2022. |
| Matteo 2024 | 10.1007/s11695-024-07109-4 | 315 | A retrospective 2-year follow-up cohort of ESG subjects was analyzed to investigate the presence of any baseline or early indicators of long-term optimal or suboptimal ESG outcomes. |
| Nigro 2025 | 10.1007/s11695-025-08155-2 | 23 | A total of 43 patients with class I and II obesity were included in the study. Twenty-three patients underwent ESG, and 20 patients were treated with Liraglutide. |
| Polese 2022 | 10.1007/s13304-022-01385-4 | 27 | 27 patients who underwent ESG were assessed for TBWL, EBWL, and ESG safety. |
| Sartoretto 2018 | 10.1007/s11695-018-3135-x | 121 | 121 patients who underwent ESG were collected for data across a 12-month follow-up. |
| Sharaiha 2021 | 10.1016/j.cgh.2020.09.055 | 216 | 216 patients who underwent ESG were analyzed for TBWL and AE across 5-years follow-up. |
| Silva 2025 | 10.1007/s11695-025-07945-y | 90 | 90 patients who underwent ESG were assessed for TBWL% and BMI. |
| Valentin Mocanu 2025 | https://doi.org/10.1007/s11695-025-07893-7 | 2285 | After identifying ESG and LSG cases using a combination of CPT and procedural code variables, non-parsimonious  multivariable logistic regression models were conducted to identify predictors of serious complications and outpatient  emergency department (ED) visits. |
| **DOI**: Digital Object Identifier, **No.**: included total number of patients, **Ref No.:** Corresponding reference number. | | | |

**Supplementary Table 2.** DOI table.

| **Study ID** | **Ethical (IRB) approval** | **Source of funding** |
| --- | --- | --- |
| Abad 2025 | This study was approved by the ethics committee/institutional review board at each site (Ramon y Cajal and Puerta de Hierro from Madrid, Marques de Valdecilla from Cantabria, and Virgen del Rocío from Sevilla). | This study was funded by a grant from the Carlos III Institute (FIS PI17/00499), Ministry of Science and Innovation of Spain. |
| Alqahtani 2022 | Ethical approval was not declared in this study | This study declared that there was no source of funding |
| Asokkumar 2023 | Ethical approval was not declared in this study | The source of funding was not declared |
| Badurdeen 2021 | Ethical approval was not declared in this study | The following authors disclosed financial relationships: A.C. Hoff: Shareholder in Hoff Medical Group. A. N. Kalloo: Founding member of, equity holder in, and consultant for Apollo Endosurgery. M. A. Khashab: Medical advisory board for Boston Scientific and Olympus America; consultant for Boston Scientific, Medtronic, and Olympus America. M. G. Neto: Consultant for Fractyl Labs, USGI, GI Dynamics, Apollo EndoSurgery, and Colubris Mx; proctor for Fractyl Labs, USGI, GI Dynamics, and Apollo EndoSurgery; scientific advisory board for GI Dynamics and Keyron (UK); trainer for Apollo EndoSurgery, Ethicon EndoSurgery, Olympus America, and Medtronic. V. Kumbhari: Consultant for Medtronic, Pentax Medical, Boston Scientific, FujiFilm, Apollo Endosurgery, and Obalon; research support from ERBE USA and Apollo Endosurgery. All other authors disclosed no financial relationships. |
| Barrichello 2019 | Institutional Review Board (IRB) approval for a retrospective analysis was obtained for each center before collecting data for this study. | The following authors disclosed financial relationships relevant to this publication: E. Guimaraes Hourneaux de Moura: Consultant for Boston Scientific and Olympus. M. Galvao dos Passos Neto: Consultant and proctor for Fractyl Labs, GI Dynamics, Apollo Endosurgery, GI Windows, Ethicon Endosurgery, Medtronics, Olympus, M.I. Tech, NitiNotes, and Alacer Biomedica. C. C. Thompson: Consultant for Boston Scientific and Medtronic; consultant and institutional grants from USGI Medical, Olympus, Fractyl Labs, and Apollo Endosurgery; ownership interest in GI Windows; research support from Aspire Bariatrics and Spatz; and consultant for and research support from GI Dynamics. All other authors disclosed no financial relationships relevant to this publication. |
| Bhandari 2020 | Ethical approval was not declared in this study | This study declared that there was no source of funding |
| Brethauer 2012 | Ethical approval was not declared in this study | All the investigators received support from C. R. Bard for pre-clinical and clinical research. |
| Carr 2022 | The study received ethical approval from the Bond University Human Research Ethics Committee (HREC) (Reference: SM02936). | This study was funded by the Commonwealth of Australia Innovation Connection grants and a grant from Weightloss Solutions Australia. |
| Castagneto-Gissey 2024 | The ethical approval of this study was waived by the Ethical Committee of Sapienza University of Rome due to its retrospective nature. Written informed consent was obtained before all endoscopic procedures. | This study declared that there was no source of funding |
| Cheskin 2020 | Ethical approval was not declared in this study | The following authors disclosed financial relationships relevant to this publication: S. Kahan: Personal fees from Novo Nordisk, Biologix, and KVK Tech. M. A. Khashab: Consultant for Boston Scientific, Olympus America, and Medtronic; advisory board for Boston Scientific and Olympus; research support from Cook Medical. A. N Kalloo: Founding member, equity holder, and consultant for Apollo Endosurgery. V. Kumbhari: Consultant for Apollo Endosurgery, Boston Scientific, Medtronic, Pentax, and ReShape Life Sciences; research support from Erbe and Apollo Endosurgery. All other authors disclosed no financial relationships relevant to this publication. |
| Correia 2023 | All procedures were performed in accordance with the ethical standards of the institutional committee and with the 1964 Helsinki Declaration and its later amendments or comparable ethical standards. Individual patient informed consent has been obtained prior to each procedure. | This study declared that there was no source of funding |
| Devière 2008 | The study was approved by the ethical committees of Erasme University Hospital (Brussels, Belgium) and Hospital Regional 1st de Octubre, Mexico City, Mexico. | This study was funded by Satiety, Inc. of Palo Alto, CA. Professor Devie`re and Dr. Edmundowicz are scientific advisors to the company, and Dr. Scott and Dr. de la Torre are co-inventors of the TOGa System and founders of the company. |
| Espinet-Coll 2022 | This study was approved by Ethical Committee of Quirón-Dexeus University Hospital (Barcelona, Spain) approved this study. | Surveillance was partially funded by a grant from Spanish Society for Gastrointestinal Endoscopy (SEED) in 2016. |
| Familiari 2011 | This study’s protocol was approved by the ethics committee of both hospitals. | P. Familiari and V. Perri received travel support from Satiety Inc. G. Costamagna and J. Devière received travel and research grant support from Satiety Inc for the completion of the study. No other financial relationships relevant to this publication were disclosed. |
| Farha 2021 | Ethical approval was not declared in this study | This study declared that there was no source of funding |
| Fayad 2019 | Ethical approval was not declared in this study | The source of funding was not declared |
| Fiorillo 2020 | All procedures performed in studies involving human participants were in accordance with the ethical standards of the institutional and/or national research committee and with the 1964 Helsinki declaration and its later amendments or comparable ethical standards. | The source of funding was not declared |
| Galva Neto 2020 | After institutional review board (IRB) approval and following the Health Insurance Portability and Accountability Act guidelines, the authors prospectively enrolled 308 patients undergoing ESG at four bariatric centers in Brazil between April 1, 2017 and December 31, 2018. | The source of funding was not declared |
| Glaysher2019 | This study was approved by the local Medical Advisory Committee | The source of funding was not declared |
| Hajifathalian 2021 | This study was approved by the IRB of the medical center of New York-Presbytarian Hospital (IRB Protocol 1510016654) | The source of funding was not declared |
| Huberty 2018 | This study was approved by the Ethical committees of Erasme Hospital, Policlinico Gemelli, and Universitè Libre ClinicalTrials.gov (NCT02582229) | This study was funded by Endotool SA (they provided instruments free of charge along with a grant) |
| James 2020 | The author declared exemption from IRB approval due to the retrospective nature of the study | This study was funded by the NIH |
| Kozlowska 2022 | This study was approved by the ethical committee of Pomeranian Medical University (IRB 3857/2022P) | The source of funding was not declared |
| Limas 2024 | This study was approved by the Ethical Committee of Sumber Waras Hospital 12/RSSW/KoM.EP/EC/X/2023. | This study declared that there was no source of funding |
| Lopez-Nava 2021 | This study was approved by the IRB (no further clarification was given) | The source of funding was not declared |
| Maselli 2023 | This study was approved by WCG IRB, Puyallup, WA | The source of funding was not declared |
| Matteo 2024 | This study was approved by the institutional ethics committee of the Digestive Endoscopy Unit of Fondazione Policlinico Universitario A. Gemelli IRCCS in Rome. (number 2083/2018) | This study was funded by pen access funding provided by Università Cattolica delSacro Cuore within the CRUI-CARE Agreement. |
| Nigro 2025 | This study was approved by the IRB of the University Hospital G. Martino of Messina | The source of funding was not declared |
| Polese 2022 | This study was approved by the IRB (no further clarification was given) | This study was funded by open-access funding provided by Università degli Studi di Padova within the CRUI-CARE Agreement. |
| Sartoretto 2018 | Ethical approval was not declared in this study | This study declared that there was no source of funding |
| Sharaiha 2021 | This study was approved by the local IRB (IRB Protocol 1510016654) 1510016654) | The source of funding was not declared |
| Silva 2025 | This study was approved by the IRBs of the participating institutions. | This study declared that there was no source of funding |
| Valentin Mocanu 2025 | This study was approved by the IRBs of the participating institutions. | This study declared that there was no source of funding |
| **IRB**: Institutional Review Board. | | |

**Supplementary Table 3.** Ethical approval and funding source for each study.

**Supplementary Figure 1.** Risk of bias assessment of randomized controlled trials using the RoB 2 tool.

|  |  |  |
| --- | --- | --- |
| (a) Post-operative bleeding | (b) Post-operative epigastric pain | (c) Vomiting |

**Supplementary Figure 2.** Funnel plots of post-operative complications.

|  | **%TBWL** | **%EBWL** | **BMI reduction** |
| --- | --- | --- | --- |
| **1 month** |  |  |  |
|  | (a) 1 month %TBWL | (b) 1 month %EBWL | (c) 1 month BMI reduction |
| **3 months** |  |  |  |
|  | (d) 3 months %TBWL | (e) 3 months %EBWL | (f) 3 months BMI reduction |
| **6 months** |  |  |  |
|  | (g) 6 months %TBWL | (h) 6 months %EBWL | (i) 6 months BMI reduction |

**Supplementary Figure 3.** Short-term continuous outcomes funnel plots

|  | **%TBWL** | **%EBWL** | **BMI reduction** |
| --- | --- | --- | --- |
| **1 year** |  |  |  |
|  | (a) 1 year %TBWL | (b) 1 year %EBWL | (c) 1 year BMI reduction |
| **2 years** |  |  |  |
|  | (d) 2 years %TBWL | (e) 2 years %EBWL |  |
| **3 years** |  |  |  |
|  | (f) 3 years %TBWL | (g) 3 years %EBWL |  |

**Supplementary Figure 4.** Long-term continuous outcomes funnel plots

|  |  |
| --- | --- |
| (a) Length of post-operative hospital stay | (b) Re-admission rate |
|  |  |
| (c) Re-operation rate | (d) Mortality rate |

**Supplementary Figure 5.** Post-operative secondary outcomes

(a) Bleeding

(b) Epigastric pain

(c) Vomiting

**Supplementary Figure 6.** Leave-one-out sensitivity analyses of post-operative complications: (a) Bleeding, (b) Epigastric pain, and (c) Vomiting.

|  | **%TBWL** | **%EBWL** | **BMI reduction** |
| --- | --- | --- | --- |
| **1month** |  |  |  |
|  | (a) 1 month %TBWL | (b) 1 month %EBWL | (c) 1 month BMI reduction |
| **3 months** |  |  |  |
|  | (d) 3 months %TBWL | (e) 3 months %EBWL | (f) 3 months BMI reduction |
| **6 months** |  |  |  |
|  | (g) 6 months %TBWL | (h) 6 months %EBWL | (i) 6 months BMI reduction |

**Supplementary Figure 7.** Leave-one-out sensitivity analyses of continuous primary short-term outcomes.

|  | **%TBWL** | **%EBWL** | **BMI reduction** |
| --- | --- | --- | --- |
| **1 year** |  |  |  |
|  | (A) 1 year %TBWL | (B) 1 year %EBWL | (C) 1 year BMI reduction |
| **2 years** |  |  |  |
|  | (D) 2 years %TBWL | (E) 2 years %EBWL |  |
| **3 years** |  |  |  |
|  | (G) 3 years %TBWL | (H) 3 years %EBWL |  |

**Supplementary Figure 8.** Leave-one-out sensitivity analyses of continuous primary long-term outcomes.

|  |  |  |
| --- | --- | --- |
| (a) Short-term %TBWL | (b) Short-term %EBWL | (c) Short-term BMI change |
|  |  |  |
| (d) Long-term %TBWL | (e) Long-term %EBWL | (f) Long-term BMI change |
|  |  |  |
| (g) Short vs long-term %TBWL | (h) Short vs long-term %EBWL | (i) Short vs long-term BMI change |

**Supplementary Figure 9.** Meta-regression bubble plots of continuous primary outcomes
